# Spheroids and organoids to study long noncoding RNAs in cancer: how they could replace animal models

**DOI:** 10.1007/s10555-026-10350-1

**Published:** 2026-06-26

**Authors:** Ana Maria Capela, João Pessoa, Hugo Arede, Bruno Bernardes de Jesus

**Affiliations:** Department of Medical Sciences and Institute of Biomedicine (iBiMED), University of Aveiro, 3810-193 Aveiro, Portugal

**Keywords:** Cancer, Spheroid, Organoid, Xenograft, Long noncoding RNA, 3D cell culture

## Abstract

Spheroids, organoids, and further three-dimensional cellular models represent an intermediate stage of supra-cellular complexity between monolayered cell cultures and animal models. In the present review, we identified conditions in which spheroids and organoids could replace animal models in biomedical research, using long noncoding RNA (lncRNA) research in cancer as a study object. In tumor spheroids and patient-derived organoids, chemosensitivity can be enhanced through therapeutic overexpression or silencing of specific lncRNAs (depending on the lncRNA and context), which then decreases spheroid/organoid size or formation efficiency. These outcomes consistently mimic observations in xenograft mouse models, in which the same lncRNA intervention decreases tumor volume. Therefore, as a proxy of tumor growth assessment, animal models could be largely replaced with spheroids or organoids, taking advantage of their inexpensiveness and patient-specific features, respectively. The use of animal models could be restricted to bystander or pleiotropic effects, such as the assessment of parameters including metastasis and survival rates.

## Introduction

Biomedical research is based on a set of research models with varying levels of complexity, including simpler ones, such as cell lines, or complex systems, such as animal models, before, and ultimately, translation to humans, if possible. Animal models properly represent the complexity of human patients, as opposed to the oversimplified nature of monolayered mammalian cell cultures. In this process, experimentation in animal models remains an essential step, despite the ethical issues, elevated costs, and facility requirements involved. As such, any strategies to decrease their usage should be considered. Three-dimensional (3D) *in vitro* cellular models, which include spheroids, organoids, “organs-on-chips”, and others, cannot fully replace animal models. Nevertheless, they provide an intermediate stage of supra-cellular complexity between monolayered two-dimensional (2D) cell cultures and animal models. Their use is key to decreasing the need for animal models in research, for which their exact potential needs to be assessed.

A general replacement of the commonly used 2D cell culture method with 3D cellular models has been recommended (Jensen & Teng, 2020). Guidelines for the replacement of animal models with organoids and “organs-on-chips” have also been proposed (Hartung & Smirnova, 2025; Park et al., 2024). Several reviews have detailed the preparation, advantages, limitations, applications, and prospective developments of 3D cellular models (mostly spheroids and organoids) in cancer and other research fields (El Harane et al., 2023; Gunti et al., 2021; Živković & Opačak-Bernardi, 2025). Nevertheless, the exact conditions (and their limitations) under which 3D cellular models can replace animal models have not been uncovered yet.

The present review identifies conditions under which spheroids and organoids could decrease the need for animal models in biomedical research. Here, we focused on the 3D cellular models that are most frequently used to study the roles of long noncoding RNAs (lncRNAs) in specific cancer types. lncRNAs are RNA molecules with a length longer than 200 nucleotides, which are not translated into proteins. Therefore, their function is directly determined by their sequence, structure, and subsequent associations with other RNAs, DNA, or proteins. The function of several lncRNAs has been detailed in several cellular processes, from cellular reprogramming to aging and cancer. Multiple lncRNAs, such as *H19 imprinted maternally expressed transcript* (*H19*), *metastasis-associated lung adenocarcinoma transcript 1* (*MALAT1*), *HOX antisense RNA* (*HOTAIR*), and *noncoding RNA activated by DNA damage* (*NORAD*), have been associated with poor prognosis in multiple cancers (Cai et al., 2014; Capela et al., 2024; Fang & Fullwood, 2016; S. Fu et al., 2020; Soghli et al., 2021; J. Yang et al., 2021). For instance, *NORAD* is upregulated in triple-negative breast cancer, and its silencing sensitizes cancer cells to chemotherapy (Alves-Vale et al., 2023; Capela et al., 2024). In the present review, we selected recently published original research articles in which at least one 3D cellular model was used to assess the impact of cellular levels of one lncRNA on the 3D model’s behavior and therapy response. Throughout this article, we use the terms “upregulation” and “downregulation” to indicate the association between 3D cellular model formation and its alterations in endogenous levels of a specific lncRNA, relative to a cognate 2D cellular model. Many of the cited studies utilized both an animal model and a 3D cellular model, in which we compared the outcomes obtained from the two models. With this approach, we uncovered conditions under which the 3D cellular model could replace the animal model.

## Spheroids: the simplest 3D cellular models

The simplest 3D cellular models are spheroids, which in the literature can also be termed as spheres or cell spheroids. When prepared from specific cell types, they may also have specific designations, such as neurospheres (generated from neuronal cells) or mammospheres (generated from breast cells). When using cancer cells, the spheroids can be termed as tumorspheres, onco-spheroids, or multicellular tumor spheroids (MCTS) (Mitrakas et al., 2023). Spheroids are prepared through *in vitro* cell growth under conditions that disable their adherence to a surface (for example, by using ultra-low adherence [ULA] cell culture plasticware), or through other methods including hanging-drop, bioprinting, or spinner flasks. Under such conditions, the cells form intercellular interactions, generating a 3D aggregate resembling a sphere (Białkowska et al., 2020; Decarli et al., 2021). Spheroid preparation is relatively simple and inexpensive. When prepared from cancer cells, this 3D system can be regarded as a simplistic mimic of a tumor (Arora et al., 2024).

Spheroids containing one or more cell types are termed homotypic or heterotypic, respectively, in which different cell types may not directly interact. In addition to the combination of different types of cells (i.e., cancer cells and cancer-associated fibroblasts [CAFs], cancer cells and immune cells, cancer cells, and endothelial cells), the complexity of spheroids can be increased by including extracellular matrix (ECM)-based biomaterials, which can promote the self-organization of the supracellular structures (Sousa et al., 2025). They can also be subjected to perfusion in microfluidic devices to study dynamic flow (to supply culture medium and remove waste products, to connect the medium—and therefore the secretome—of different wells growing different cells, among other goals) (Białkowska et al., 2020). The potential of this model is due to its versatility for customization, as exemplified above. Furthermore, spheroids can be used for testing the effects of drugs and alterations in lncRNA levels, and they can be personalized by using patient-derived cells. Table 1 summarizes lncRNA studies in spheroids. Collectively, these studies show that spheroids can have different levels of specific lncRNAs relative to the same cell line cultured in a 2D monolayer. Identical alterations could be observed in patient tissues relative to healthy individuals. They also demonstrate that lncRNAs are convenient molecules that modulate spheroid formation in multiple models of cancer.

**Table 1:** Studies that use spheroids to study lncRNAs

Cancer type	lncRNA	Summary	Ref
Adrenocortical carcinoma	H19 imprinted maternally expressed transcript (H19)	*H19* was ↓ expressed in adrenocortical carcinoma. Silencing *H19* with siRNAs did not impact spheroid growth, but deacetylase inhibitors ↓ spheroid growth and ↑ *H19* expression	(Di Fazio et al., 2022)
Bladder cancer	FOXF1 adjacent non-coding developmental regulatory RNA (FENDRR)	*FENDRR* was ↓ expressed in bladder cancer. *FENDRR* overexpression ↓ spheroid formation efficiency	(Dai et al., 2025)
	Metastasis associated lung adenocarcinoma transcript 1 (MALAT1)	*MALAT1* was ↑ expressed in “leader” cells of patient-derived spheroids	(Li et al., 2024c)
Breast cancer	AFAP1 antisense RNA 1 (AFAP1-AS1)	*AFAP1-AS1* was ↑ expressed in breast cancer tissues. AFAP1-AS1 silencing in spheroids grown in Matrigel ↓ hypoxia-induced 3D channel formation	(García-Hernández et al., 2024)
	Deleted in lymphocytic leukemia 2 (DLEU2)	*DLEU2* was ↑ expressed in breast cancer cell lines and tissues, and was also ↑ expressed in spheroids. *DLEU2* silencing ↓ spheroid formation efficiency	(Islam et al., 2024)
	H19 imprinted maternally expressed transcript (H19)	*H19* expression in breast cancer was associated with poor prognosis. Silencing *H19* ↓ spheroid formation efficiency	(Shima et al., 2018)
	Long intergenic non-protein coding RNA 52 (LINC00052)	*LINC00052* was ↓ expressed in spheroids	(Muñoz-Galindo et al., 2019)
	Long intergenic non-protein coding RNA 511 (LINC00511)	Knockout of *LINC00511* ↓ spheroid numbers, dimension and formation frequency number, as well as stemness levels	(Azadbakht et al., 2022)
		*LINC00511* was ↑ expressed in breast cancer tissues. *LINC00511* overexpression ↑ the diameter of spheroids and stemness levels, and accelerated proliferation and invasion. Mutating *LINC00511* (which ↓ the amount of wild-type *LINC00511*) ↓ spheroid diameter, stemness, proliferation and migration, and promoted apoptosis	(Tan et al., 2023)
	Long intergenic non-protein coding RNA 520 (LINC00520)	*LINC00520* was ↑ expressed in human tumors. Silencing *LINC00520* ↓ the number of cell protrusions and cell migration of spheroids in Matrigel	(Henry et al., 2016)
	Long intergenic non-protein coding RNA 885 (LINC00885)	*LINC00885* was ↑ expressed in breast ductal carcinoma. Overexpressing LINC0085 in normal breast cells ↑ spheroid growth and branching	(Abba et al., 2020)
	RP11-20F24.2 [ENSG00000234918]; SKAP1 antisense RNA 2 (SKAP1-AS2); RP11-206M11.7 [ENSG00000244468.1]; CTD-2566J3.1 [ENSG00000258837.1]; urothelial cancer associated 1 (UCA1); LASP1 neighbor (LASP1NB); long intergenic non-protein coding RNA 1535 (LINC01535); CTB-119C2.1 [ENST00000608362.1]; MIR924 host gene (MIR924HG); PRRT3 antisense RNA 1 (PRRT3-AS1); X inactive specific transcript (XIST); RP11-782C8.3 [ENSG00000232274.2]; cytoskeleton regulator RNA (CYTOR); MIR4435-2 host gene (MIR4435-2HG); CTD-2538C1.2 [ENSG00000267475.1]; long intergenic non-protein coding RNA 857 (LINC00857); RP11-425M5.7 [ENSG00000276603]; BLCAP apoptosis inducing factor antisense 1 (BLCAP-AS1); RP11-383J24.6 [ENSG00000237586]	Extracellular matrix protein-enriched 3D cultures were used to identify differentially expressed lncRNAs. The identified ↑ expressed lncRNAs were as follows: *RP11-20F24.2*,* SKAP1-AS2*,* also known as THRA1/BTR*,* RP11-206M11.7*,* CTD-2566J3.1*,* UCA1*,* LASP1NB* also known as *LINC00672*,* LINC01535*,* CTB-119C2.1 MIR924HG*, and *PRRT3-AS1*. The identified ↓ expressed lncRNAs were as follows: *XIST*,* RP11-782C8.3*,* CYTOR* also known as *LINC00152*,* MIR4435-2HG*,* CTD-2538C1.2*,* LINC00857*,* RP11-425M5.7*,* BLCAP-AS1*, and *RP11-383J24.6*	(Nuñez-Olvera et al., 2022)
Cervical cancer	AFAP1 antisense RNA 1 (AFAP1-AS1)	CD44v6+ cells, which express more stemness-related genes, formed more and larger spheroids than CD44v6-. Silencing *AFAP1-AS1* ↓ spheroid number and size	(Xia et al., 2021)
	WWTR1 antisense RNA 1 (WWTR1-AS1)	*WWTR1-AS1* was ↑ expressed in cervical squamous cell carcinoma tissues. Overexpressing *WWTR1-AS1* ↑ spheroid formation and the amount of CD133+ cells	(Zhou et al., 2024)
	HOXA cluster antisense RNA 2 (HOXA-AS2), urothelial cancer associated 1 (UCA1), HOX transcript antisense RNA (HOTAIR), HIF1A antisense RNA 1 (HIF1A-AS1), imprinted in Prader-Willi syndrome (IPW)	Spheroids were more resistant to cisplatin than 2D monolayer cells. Cisplatin treatment in secondary spheroids (which were fist cultured in spheroids, then in a 2D monolayer, and later in spheroids) ↓ secondary spheroid formation enriched in CSCs. *HOXA-AS2*,* UCA1*,* HOTAIR*,* HIF1A-AS1* and *IPW* were ↑ expressed in cisplatin-treated spheroids. Downregulating *HOXA-AS2*,* UCA1*,* HOTAIR*,* HIF1A-AS1* and *IPW* ↓ spheroid formation, thus enhancing the effect of cisplatin	(M. Li et al., 2019)
Cholangiocarcinoma	Lon noncoding RNA Polycystic Kidney Disease 2-2-3 (Lnc-PKD2-2–3)	*Lnc-PKD2-2–3* was ↑ expressed in CCA tissues. In DAVID bioinformatics, expression of “lnc-PKD2-2–3 was correlated with several oncogenes and stemness-associated pathways, including DGAT2, GPAM, HEPACAM, ATP1A1, PAFAH1B3, LOC728342, STAB2, SH2D3A and GTF2I”. Overexpressing Lnc-PKD2-2-3 led to ↑ number of spheroids	(Qiu et al., 2019)
Colorectal cancer	DLGAP1 antisense RNA 2 (DLGAP1-AS2)	*DLGAP1-AS2* was ↑ expressed in CD133+ rectal cancer stem cells. *DLGAP1-AS2* silencing ↓ spheroid diameter	(Xiao et al., 2022)
	H19 imprinted maternally expressed transcript (H19)	*H19* was ↑ expressed in colorectal cancer tissues. Silencing *H19* ↓ the number of formed spheroids, while leukemia inhibitory factor ↑ the spheroid count	(M. Zhu et al., 2024)
	Hypoxia-inducible factor-2α promoter upstream transcript (HIF2PUT)	Silencing *HIF2PUT* ↓ spheroid formation and the self-renewal capability of cells	(Yao et al., 2015)
	Long intergenic non-protein coding RNA 1315 (LINC01315)	Silencing *LINC01315* ↓ spheroid formation, and cultivation with CD133+/CD44+ cancer stem cell (CSC)-derived exosomes ↑ spheroid formation. However, simultaneous silencing of LINC01315 and CSC exosome cultivation reversed the effect of the exosomes on spheroid growth	(Li, et al., 2022d)
	Metastasis associated lung adenocarcinoma transcript 1 (MALAT1)	*MALAT1* was ↑ expressed in colorectal cancer cells with high CD95 levels. Silencing of MALAT1 ↓ spheroid formation	(Gao et al., 2024)
	TINCR ubiquitin domain containing (TINCR)	Overexpression of *TINCR* ↑ radioresistance of spheroids, while silencing *TINCR* had the opposite effect	(Kang et al., 2019)
	TMPO antisense RNA 1 (TMPO-AS1)	*TMPO-AS1* was ↑ expressed in colorectal cancer cells. Silencing TMPO-AS1 ↓ spheroid formation efficiency	(Ye et al., 2022)
Endometrial cancer	Long intergenic non-protein coding RNA, regulator of reprogramming (LINC-ROR)	↑ *LINC-ROR* expression was associated with the pluripotent state of endometrial cancer tissues. *LINC-ROR* silencing ↓ spheroid differentiation	(Zhou et al., 2014)
Esophageal squamous cell carcinoma	Protein disulfide isomerase family A member 3 pseudogene 1 (PDIA3P1)	Silencing *PDIA3P1* ↓ spheroid formation and size, while *PDIA3P1* overexpression had the opposite effects	(T. Huang et al., 2024)
	(Sex determining region Y)-box 2 overlapping transcript (SOX2OT)	*SOX2OT* was ↑ expressed in esophageal squamous cell carcinoma tissues and spheroids, as compared to 2D monolayer cells. *SOX2OT* silencing ↓ spheroid number and size, and ↑ sensibility to docetaxel treatment	(Haghi et al., 2022)
Gastric cancer	ADAMTS9 antisense RNA 2 (ADAMTS9-AS2)	*ADAMTS9-AS2* was ↓ expressed in gastric cancer tissues. *ADAMTS9-AS2* overexpression ↑ spheroid number	(F. Wang et al., 2020)
Glioma	Long intergenic non-protein coding RNA, regulator of reprogramming (LINC-ROR)	*LINC-ROR* was ↓ expressed in glioma tissues. Overexpressing *LINC-ROR* ↓ spheroid number, while *LINC-ROR* silencing had the opposite effect	(Feng et al., 2015)
	Nuclear paraspeckle assembly transcript 1 (NEAT1)	*NEAT1* was ↑ expressed in glioblastoma multiform spheroids	(Yoon et al., 2020)
	Prostate cancer associated transcript 1 (PCAT1)	*PCAT1* was ↑ expressed in glioma CSCs. Silencing *PCAT1* ↓ spheroid formation, while *PCAT1* overexpression had the opposite effect	(Zhang et al., 2019a)
	Small nucleolar RNA host gene 9 (SNHG9)	*SNHG9* was ↑ expressed in glioma stem cells, as compared to glioma cells. *SNHG9* overexpression ↑ spheroid formation efficiency, while *SNHG9* silencing had the opposite effect	(Wang et al., 2022a)
	HOXD cluster antisense RNA 2 (HOXD-AS2), Long intergenic non-protein coding RNA 1116 (LINC01116)	*HOXD* was ↑ expressed in glioma cells and glioblastoma tissues. Silencing *HOXD-AS2* or *LINC01116* ↓ spheroid formation	(Deforzh et al., 2022)
Hepatocellular carcinoma	ICAM4 antisense RNA 1 (ICAM4-AS1)	*ICAM4-AS1* (also known as *ICR*) was ↑ expressed in PVTT (portal vein tumor thrombus, common in liver cancer) tissues and hepatocellular carcinoma cell lines. Silencing *ICAM4-AS1* ↓ spheroid formation	(W. Guo et al., 2016)
Lung cancer	Differentiation antagonizing non-protein coding RNA (DANCR)	*DANCR* was ↑ expressed in non-small cell lung cancer (NSCLC). Silencing *DANCR* ↓ spheroid size	(Nicolescu et al., 2022)
	HOX transcript antisense RNA (HOTAIR)	*HOTAIR* was ↑ expressed in a wide variety of cancers, including lung cancer. Collagen I supplementation in lung cancer spheroids ↑ *HOTAIR* expression	(Y. Zhuang et al., 2013)
		*HOTAIR* was ↑ expressed in cisplatin-resistant groups of patients and in cisplatin-resistant cell lines, and also correlated with the pathological grade. Overexpressing *HOTAIR* ↑ spheroid formation, while silencing *HOTAIR* had the opposite effect	(M. Y. Liu et al., 2016)
	JPX (just proximal to XIST) transcript, XIST activator (JPX)	*JPX* was ↑ expressed in lung adenocarcinoma tissues. Overexpressing JPX ↑ the number of cells in spheroids	(Mosca et al., 2024)
	lncRNA-AC026356.1 [ENSG00000274964]	*lncRNA-AC026356.1* was ↑ expressed in chemo-resistant lung cancer cells, and in spheroids. *lncRNA-AC026356.1* silencing ↓ spheroid number	(Zhang et al., 2023c)
	Long intergenic non-protein coding RNA 1224 (LINC01224)	*LINC01224* was ↑ expressed in irradiation-resistant NSCLC cells. *LINC01224* silencing ↓ spheroid formation	(Fu et al., 2021b)
	Long intergenic non-protein coding RNA 1232 (LINC01232)	*LINC01232* was ↑ expressed in NSCLC cells. Silencing *LINC01232* ↓ spheroid number and formation	(L. Zhu et al., 2022)
	PITPNA antisense RNA 1 (PITPNA-AS1)	*PITPNA‐AS1* was ↑ expressed in lung squamous cell carcinoma . *PITNAS-AS1* overexpression ↑ spheroid formation	(B. hao Peng et al., 2022)
Melanoma	JUN inducer (JUNI) or JUN divergent transcript (JUN DT)	*JUNI* was ↑ expressed in cancer cells after stress induction. Doxorubicin treatment ↓ cell survival of *JUNI*-silenced spheroids	(Kumar et al., 2024)
Osteosarcoma	Hypoxia-inducible factor-2α promoter upstream transcript (HIF2PUT)	*HIF2PUT* expression was correlated with stem cell-like properties and CSCs of tumors. Silencing *HIF2PUT* ↑ spheroid formation rate	(Y. Wang et al., 2015)
Ovarian cancer	HLA complex P5 (HCP5)	*HCP5* knockout ↓ spheroid formation and size	(Moradi et al., 2024)
	Long intergenic non-protein coding RNA 958 (LINC00958)	*LINC00958* was ↑ expressed in ovarian cancer cells. Cells with higher basal levels of *LINC0095*8 had ↑ sphere formation ability; however, *LINC00958* silencing reversed this increase. Co-culture with exosomes from the cell line with the highest *LINC00958* expression also ↑ the spheroid formation ability of the cells with lowest *LINC00958* expression. However, silencing *LINC00958* in the highest *LINC00958* expressing cell line reverted the previous effect	(Yan et al., 2024)
Pancreatic cancer	Long stress-induced non-coding transcript 5 (LSINCT5)	Patients with low *LSINCT5* had ↑ survival rates. *LSINCT5* knockout reduced spheroid formation, number, and size	(Y. Dai et al., 2020)
Papillary thyroid carcinoma	H19 imprinted maternally expressed transcript (H19)	*H19* was ↑ expressed in papillary thyroid carcinoma stem cells and papillary thyroid carcinoma tissues. *H19* silencing ↓ spheroid formation, while *H19* overexpression had the opposite effect	(Li et al., 2018a)
Prostate cancer	H19 imprinted maternally expressed transcript (H19)	*H19* was ↑ expressed in prostate cancer cells enriched in stem cell properties, and even more ↑ expressed in prostate cancer cell lines and other cancer cell lines. Overexpression of *H19* ↑ spheroid number and size, while *H19* silencing had the opposite effect	(Roy et al., 2015)
	NUTM2A antisense RNA 1 (NUTM2A-AS1)	*NUTM2A-AS1* was ↑ expressed in NSCLC and prostate cancer. Silencing *NUTM2A-AS1* ↓ spheroid number	(H. Han et al., 2024)

The molecular mechanisms that mediate spheroid formation through alterations in lncRNA levels are starting to be uncovered. In general, increased spheroid formation is correlated with increased stemness, which could be a driving force. For example, cytoplasmic lncRNA *H19* has oncogenic properties in several cancer types, associated with increased stemness, through mechanisms that involve microRNA inhibition (J. Yang et al., 2021). In papillary thyroid carcinoma, *H19* expression enhances cancer stem-like properties (Li et al., 2018a). In breast cancer, *H19* inhibits the let-7 microRNA, causing an increase in the levels of its target, the LIN28 RNA-binding protein (F. Peng et al., 2017). In prostate cancer, silencing of *H19* decreased both spheroid formation and the expression of the stemness markers octamer-binding transcription factor 4 and SRY-box transcription factor 2 (Roy et al., 2015). *Long intergenic non-protein coding RNA, regulator of reprogramming (LINC-ROR)*, also located primarily in the cytoplasm, was upregulated in esophageal squamous cell carcinoma and positively correlated with stemness marker SRY-box transcription factor 9; their interaction was mediated by microRNAs (L. Wang et al., 2017). In endometrial cancer stem cells, *LINC-ROR* was also positively correlated with stemness. The miR-145 microRNA could downregulate *LINC-ROR* and inhibit spheroid formation (Zhou et al., 2014). However, *LINC-ROR* was downregulated in glioma tissues, and its expression was negatively correlated with the stemness marker Krüppel-like factor 4 (Feng et al., 2015). For this lncRNA, increased stemness was also correlated with increased spheroid formation.

*HOTAIR*, which functions in both the cytoplasm and nucleus, is upregulated in colorectal cancer, where its silencing inhibits stemness and tumorigenicity by inhibiting the miR-211-5p microRNA and upregulating the fms-like tyrosine kinase-1 (Y. Huang et al., 2021. In non-small cell lung cancer, both *HOTAIR* and stemness markers were upregulated (M. Y. Liu et al., 2016). In ovarian cancer, *HOTAIR* maintains stemness by targeting the miR-206 microRNA and upregulating the T-box transcription factor 3 (Y. Zhang et al., 2020). *HOTAIR* is another lncRNA whose upregulation increases both stemness and spheroid formation. Additional lncRNAs have been shown to promote stemness and spheroid formation, such as *MALAT1*, a predominantly nuclear lncRNA, that enhances triple-negative breast cancer tumorigenesis and stemness by downregulating DNA methyltransferase 1 through the miR-137 microRNA and the B-cell lymphoma/leukemia 11 A transcription factor (Hu et al., 2023). These findings indicate that the *H19*, *LINC-ROR*, *MALAT1*, and *HOTAIR* lncRNAs promote spheroid formation by enhancing stemness through gene regulation networks involving microRNA inhibition and protein upregulation.

Spheroids can have reduced sensitivity to chemotherapeutic drugs, relative to identical 2D-cultured cells. For example, cervical cancer spheroids present a higher resistance to cisplatin treatment than the same 2D-cultivated cells. In comparison with 2D-cultured cells, cervical cancer spheroids had increased levels of lncRNAs, including *homeobox A cluster antisense RNA 2 (HOXA-AS2), urothelial carcinoma associated 1* (*UCA1*), *HOTAIR*, *hypoxia-inducible factor 1 alpha antisense RNA 1* (*HIF1A-AS1*), and *imprinted in Prader-Willi syndrome* (*IPW*). Silencing of these lncRNAs enhanced the cytotoxicity of cisplatin, resulting in decreased spheroid formation (M. Li et al., 2019). Furthermore, in melanoma, silencing of *JUN inducer* (*JUNI*) increased spheroid sensitivity to doxorubicin (Kumar et al., 2024). In esophageal squamous cell carcinoma, the *SOX2 overlapping transcript* (*SOX2OT*) lncRNA was upregulated in spheroids, relative to 2D cultures. *SOX2OT* silencing decreased the number and size of spheroids, where it enhanced docetaxel toxicity (Haghi et al., 2022). These examples demonstrate that the therapeutic silencing of lncRNAs, whose upregulation is associated with enhanced spheroid formation, may enhance their sensitivity to chemotherapeutic agents. Such enhanced sensitivity can be generally assessed by monitoring any decrease in spheroid size.

Alterations of the lncRNA levels can also change the differentiation stage of spheroids. For example, spheroids, generated from patient’s endometrial cancer samples cultured in stem cell medium and differentiated upon basic fibroblast growth factor (bFGF) depletion, had lower levels of *LINC-ROR.* On the contrary, overexpression of *LINC-ROR* inhibited spheroid differentiation (Zhou et al., 2014).

Spheroids can be dissociated and re-assembled in secondary spheroids. Spheroid formation is widely used to generate cellular models enriched in cancer stem cells (CSCs). Consequently, they can be used to infer self-renewal capabilities and stemness. For example, in esophageal squamous cell carcinoma, some stemness markers were upregulated in spheroids (Haghi et al., 2022). In addition, both the size and number of breast cancer spheroids were increased in the presence of the stemness marker leukemia inhibitory factor (M. Zhu et al., 2024). Therefore, studying spheroid formation and growth is also useful to assess the stemness properties of cancer cells.

In addition to lncRNA silencing, spheroid size can be altered by treatment with specific small molecules, or lncRNA-interacting proteins, resulting in similar effects on spheroid size. Treatment of adrenocortical carcinoma spheroids with deacetylase inhibitors induced *H19* overexpression and reduced their size (Di Fazio et al., 2022). Spheroid formation can also be manipulated by adding complex mixtures that affect lncRNA expression. In ovarian cancer, the secretome of mesenchymal stem cells (MSCs) derived from the amniotic fluid or the chorionic villi caused a shift in lncRNA expression and decreased spheroid formation (Vaiasicca et al., 2024). These approaches provide alternative ways to influence lncRNA expression and effects on spheroid formation and size.

### Cancer spheroids can mimic *ex vivo* tumor growth

Multiple studies have combined cancer cell spheroids with xenografts in mouse models, showing consistent results in their responses to lncRNA manipulation. Specific examples of studies that have been published in several cancer types are summarized in Table 2. Overall, these studies indicate that tumor growth can be correlated, and even represented, by spheroid growth, in multiple cancer types. Their global analysis supports that *in vitro* spheroids could robustly mimic *in vivo* tumors regarding their response to the manipulation of lncRNA levels. Nevertheless, properties including metastasis and animal survival still require the use of animal models. Further research is needed to determine the parallelism between these models and how they can accurately represent time and dose-response in cancer. It is expected that study complementation with these 3D models may decrease the number of animals needed and restrict the use of animal models to properties that cannot be mimicked *in vitro*.

**Table 2:** Studies that use spheroids and xenografts to study lncRNAs

Cancer type	lncRNA	Summary	Ref
Bladder cancer	Brain cytoplasmic RNA 1 (BCYRN1)	*BCYRN1* seemed ↑ expressed in bladder cancer. *BCYRN1* silencing ↓ spheroid formation, as well as ↓ tumor weight and tumor volume in xenografted mice	(Arima et al., 2024)
Breast cancer	Colon cancer-associated transcript 2 (CCAT2)	*CCAT2* was ↓ expressed in tumor tissues from luminal breast cancer patients. *CCAT2* overexpression ↓ the number and diameter of spheroids, and the diameter of tumors in xenografted mice	(Xie et al., 2023)
	Differentiation antagonizing non-protein coding RNA (DANCR)	*DANCR* was ↑ expressed in triple negative breast cancer cell lines. Silencing of *DANCR* ↓ the size of the networks formed by spheroids growing in Matrigel and the size of tumors in xenografted mice	(Nicolescu et al., 2023)
	H19 imprinted maternally expressed transcript (H19)	*H19* was ↑ expressed in breast cancer tumors and cell lines. *H19* overexpression ↑ the number and diameter of spheroids, while *H19* silencing ↓ tumor volume in xenografted mice	(F. Peng et al., 2017)
		*H19* was ↑ expressed in breast cancer xenograft tumors and cell lines. Silencing of *H19* ↓ the number and diameter of spheroids, while *H19* overexpression ↑ tumor volume in xenografted mice	(F. Peng et al., 2018)
	HOX transcript antisense RNA (HOTAIR)	*HOTAIR* was ↑ expressed in breast cancer tissues. *HOTAIR* silencing ↓ spheroid formation and tumor size in xenografted mice, and ↑ survival rates of xenografted mice	(R. Han et al., 2022)
	Long intergenic non-protein coding RNA 52 (LINC00052)	*LINC00052* was ↓ expressed in patients with worse prognosis. *LINC00052* expression ↓ with spheroid formation. *LINC00052* silencing did not affect spheroid formation, but ↑ cell migration in a zebrafish xenograft model	(Sanchez-Lopez et al., 2021)
	Long intergenic non-protein coding RNA 3000 (LINC03000)	*LINC03000*, also known as *LncMat2B* was ↑ expressed in cisplatin-resistant breast cancer cells. *LINC03000* overexpression ↓ spheroid growth and survival rates in zebrafish	(Garcia-Venzor et al., 2020)
	MBNL1 antisense RNA 1 (MBNL1-AS1)	*MBNL1-AS1* was ↓ expressed in breast cancer tissues and cell lines. ↓ expression of *MBNL1-AS1* was associated with ↓ survival rates in patients. *MBNL1-AS1* silencing ↑ spheroid number and diameter, and tumor size in xenografted mice, while its overexpression had the opposite effects	(Ding et al., 2022)
	Metastasis associated lung adenocarcinoma transcript 1 (MALAT1)	*MALAT1* was ↑ expressed in triple-negative breast cancer tissues and cell lines. Silencing *MALAT1* ↓ spheroid formation and tumor volume in xenografted mice, while *MALAT1* overexpression ↑ tumor volume in this mouse model	(Hu et al., 2023)
	TOMM22 divergent transcript (TOMM22-DT)	*TOMM22-DT*, also known as *Lnc408*, was ↑ expressed in breast cancer stem cells. Silencing of *TOMM22-DT* ↓ the number of spheroids and tumor volume in xenografted mice, while *TOMM22-DT* overexpression had the opposite effects	(Wen et al., 2021)
	X inactive specific transcript (XIST)	*XIST* was ↑ expressed in breast cancer cell lines. *XIST* silencing ↓ the number of spheroids and their size, as well as tumor volume in xenografted mice	(Ma et al., 2023)
Colorectal cancer	B4GALT1 antisense RNA 1 (B4GALT1-AS1)	*B4GALT1-AS1* was ↑ expressed in colon cancer cell lines. *B4GALT1-AS1* silencing ↓ the number of spheroids and the size of tumors in xenografted mice	(Y. Zhang et al., 2019a, b)
	BACE1 antisense RNA (BACE1-AS)	*BACE1-AS* was ↑ expressed in colorectal cancer tissues and high expression of *BACE1-AS* was associated with ↓ patient survival rates. *BACE1-AS* overexpression ↑ the number and diameter of spheroids. *BACE1-AS* knockout ↓ the number and diameter of spheroids, as well as the number of metastatic foci in the livers of xenografted mice	(Wang et al., 2023b)
	Breast cancer antiestrogen resistance 4 (BCAR4)	*BCAR4* was ↑ expressed in colorectal cancer tumors. Overexpression of *BCAR4* ↑ sphere formation efficiency. In xenografted mice, *BCAR4* silencing ↓ tumor weight and volume	(Ouyang et al., 2019)
	CERS6 antisense RNA 1 (CERS6-AS1)	*CERS6-AS1* was ↑ expressed in colorectal cancer tissues. *CERS6-AS1* silencing ↓ spheroid formation efficiency and tumor weight in xenografted mice	(Zhao et al., 2022)
	GATA2 antisense RNA 1 (GATA2-AS1)	*GATA2-AS1* was ↑ expressed in colon adenocarcinoma tissues. Silencing of *GATA2-AS1* ↓ the number of spheroids, as well as the tumor weight and volume in xenografted mice	(Pan et al., 2022)
	HOX transcript antisense RNA (HOTAIR)	*HOTAIR* was ↑ expressed in colorectal cancer tumors, and high expression of *HOTAIR* was associated with ↓ patient survival rates. *HOTAIR* silencing ↓ spheroid formation and tumor volume in xenografted mice	(Y. Huang et al., 2021)
	Long intergenic non-protein coding RNA 1315 (LINC01315)	*LINC01315* was ↑ expressed in colon adenocarcinoma tissues. *LINC01315* silencing ↓ the number of formed spheroids, and tumor size and weight in xenografted mice. *LINC01315* overexpression ↑ spheroid formation	(Li et al.., 2022c)
	Pvt1 oncogene (PVT1)	*PVT1* was ↑ expressed in colon cancer tissues and high expression of *PVT1* was associated with ↓ patient survival rates. *PVT1* overexpression ↑ the number of spheroids and tumor volume in xenografted mice	(Lai et al., 2021)
	Testis development related 1 (TDRG1)	*TDRG1* was ↑ expressed in colorectal cancer cells. Silencing of *TDRG1* ↓ spheroid size and number, as well as tumor formation in xenografted mice	(Hong et al., 2022)
Cutaneous squamous cell carcinoma	Pvt1 oncogene (PVT1)	*PVT1* was ↑ expressed in cutaneous squamous cell carcinoma tissues. *PVT1* knockout ↓ spheroid area, as well as the volume and senescence levels in xenografted mouse tumors	(Li et al., 2024b)
Esophageal squamous cell carcinoma	Long intergenic non-protein coding RNA, regulator of reprogramming (LINC-ROR)	*LINC-ROR* was ↑ expressed in esophageal squamous cell carcinoma tissues. *LINC-ROR* silencing, as well as cisplatin, ↓ spheroid formation, as well as tumor mass and volume in xenografted mice	(L. Wang et al., 2017)
Gastric cancer	ASB16 antisense RNA 1 (ASB16-AS1)	*ASB16-AS1* was ↑ expressed in gastric cancer tissues and cells. *ASB16-AS1* silencing ↓ spheroid formation efficiency, as well as tumor weight and volume in xenografted mice	(Fu et al., 2021a)
	Long intergenic non-protein coding RNA 3145 (LINC03145)	*LINC03145*, also known as *CRART16*, was ↑ expressed in gastric cancer tissues. *LINC03145* overexpression ↑ number and sprouting of spheroids, tumor size in xenografted mice, and microvessel formation in chick chorioallantoic membrane assays	(Zhang et al., 2022a)
	Long intergenic non-protein coding RNA 924 (LINC00924)	*LINC00924* was ↑ expressed in gastric cancer tissues, and high *LINC00924* expression was associated with lower survival rates of patients. *LINC00924* overexpression ↑ spheroid formation and peritoneal metastasis in mice. *LINC00924* silencing ↓ spheroid formation and prevented tumor growth in xenografted mice	(He et al., 2022b)
	WT1 antisense RNA (WT1-AS)	*WT1-AS* was ↓ expressed in gastric cancer stem cells as compared to non-stem gastric cancer cells. *WT1-AS* overexpression ↓ spheroid size and tumor size in xenografted mice. Overexpression of *WT1-AS* ↓ tumor growth, stemness, and metastasis in mouse xenografts	(Zhang et al., 2023b)
Glioma	FOXD2 adjacent opposite strand RNA 1 (FOXD2-AS1)	*FOXD2-AS1* was ↑ expressed in glioma tissues, and ↑ levels of *FOXD2-AS1* were associated with ↓ patient survival rates. Silencing of *FOXD2-AS1* ↓ spheroid formation and tumor weight in xenografted mice	(Wang et al., 2022c)
	Hepatocellular carcinoma up-regulated long non-coding RNA (HULC)	*HULC* was ↑ expressed in glioblastoma tissues. *HULC* silencing ↓ spheroid formation, as well as tumor weight and volume in xenografted mice. *HULC* overexpression ↑ tumor weight in xenografted mice	(Li et al., 2022b)
	Nuclear paraspeckle assembly transcript 1 (NEAT1)	*NEAT1* was ↑ expressed in glioma cells upon infection with adenovirus. Silencing of *NEAT1* ↓ the number of spheroids. Injection of spheroids in mice was associated with larger tumors, as compared to the injection of 2D-cultured cells. Inoculation of mice with higher numbers of cells in spheroids also correlated with ↓ survival rates	(Zang et al., 2020)
	Small nucleolar RNA host gene 10 (SNHG10)	*SNHG10* was ↑ expressed in glioma cells. Silencing of *SNHG10* ↓ spheroid formation efficiency, as well as tumor volume and weight in xenografted mice	(Jin et al., 2020)
Head and neck squamous cell carcinoma	Ventricular heart development associated lncRNA (VHRT)	*VHRT*, also known as *MASCC1*, was differently expressed in metastatic head and neck squamous cell carcinoma, as compared to non-metastatic tissues of the same cancer type. Low *VHRT* expression was associated with ↑ patient survival. Silencing of *VHRT* ↓ spheroid weight and volume, as well as proliferation markers in xenografted mouse tumors. Overexpression of *VHRT* ↑ spheroid formation, as well as tumor formation and lymph node metastasis in xenografted mice	(Wang et al., 2023c)
Hypopharyngeal squamous cell carcinoma	Long intergenic non-protein coding RNA 662 (LINC00662)	*LINC00662* was ↑ expressed in hypopharyngeal squamous cell carcinoma tissues, and patients with high expression of *LINC00662* had ↓ survival rates. *LINC00662* overexpression promoted spheroid formation, and ↑ tumor volume in xenografted mice	(B. Zhang & Ye, 2024)
Liver cancer	FGD5 antisense RNA 1 (FGD5-AS1)	*FGD5-AS1* was ↑ expressed in hepatocellular carcinoma tissues and cell lines, being correlated with tumor stage and size. Patients with higher *FGD5-AS1* expression showed ↓ survival. *FGD5-AS1* silencing ↓ spheroid diameter and volume, as well as the weight, stemness, and proliferation of xenografted mouse tumors. *FGD5-AS1* overexpression ↑ spheroid diameter	(He et al., 2022a)
	FRMD6 antisense RNA 1 (FRMD6-AS1)	*FRMD6-AS1* was ↑ expressed in hepatocellular carcinoma tissues and cell lines. High expression of *FRMD6-AS1* was correlated with ↓ survival of patients. Overexpression of *FRMD6-AS1* ↑ spheroid formation and tumor size and weight in xenografted mice	(Sun et al., 2023)
	KCNQ1 opposite strand/antisense transcript 1 (KCNQ1OT1)	*KCNQ1OT1* was ↑ expressed in hepatocellular carcinoma tissues, and high expression of *KCNQ1OT1* was correlated with lower patient survival rates. *KCNQ1OT1* knockout ↓ the number of spheroids and tumor volume in xenografted mice	(Majumdar et al., 2024)
	Lipoprotein(a) like 2 (pseudogene) (LPAL2)	*LPAL2* was ↓ expressed in hepatocellular carcinoma tissues and CD133+ cells Low expression of *LPAL2* was correlated with lower patient survival. *LPAL2* silencing accelerated spheroid formation and ↑ spheroid size, as well as tumor growth and weight in xenografted mice	(Lin et al., 2022)
	lncRNA regulator of Akt signaling associated with HCC and RCC (LNCARSR)	*LNCARSR*, also known as *LncRNA ARSR*, was ↑ expressed in cisplatin-resistant hepatocellular carcinoma, in CSCs from primary liver cultures, and in spheroids derived from human primary hepatocellular carcinoma cells, relative to 2D cultures. When plated again in 2D-settings, the CSCs from trypsinized spheroids had ↓ expression of *LNCARSR*. Silencing of *LNCARSR* ↓ spheroid number and size, and resulted in a smaller population of CSC in hepatome cells when injected into mice. *LNCARSR* was also associated with chemoresistance in patients	(Yang et al., 2019a, b)
	Long intergenic non-protein coding RNA 261 (LINC00261)	*LINC00261* was ↓ expressed in TGFB1-induced epithelial-mesenchymal transition hepatocellular carcinoma cell lines. Overexpression of *LINC00261* ↓ spheroid number and size and ↓ tumor weight and growth rate in xenografted mice	(Chen & Xiang, et al., 2022b)
	Long intergenic non-protein coding RNA 680 (LINC00680)	*LINC00680* was ↑ expressed in hepatocellular carcinoma tissues and cell lines, was correlated with the tumor stage and size, and patients with high expression of *LINC00680* had lower survival rates. Overexpression of *LINC00680* ↑ primary and secondary spheroid formation. Silencing *LINC00680* ↓ primary and secondary spheroid formation, and, in xenografted mice, modified stemness markers and enhanced antitumor potential of the drug tested	(Shu et al., 2021)
	OIP5 antisense RNA 1 (OIP5-AS1)	*OIP5-AS1* was ↑ expressed in hepatoblastoma cells. Silencing *OIP5-AS1* ↓ the number of spheroids and tumor weight in xenografted mice	(W. Jiang et al., 2022)
	Small nucleolar RNA host gene 3 (SNHG3)	*SNHG3* was ↑ expressed in hepatocellular carcinoma primary tumors. *SNHG3* silencing ↓ spheroid formation efficiency and ↑ survival rates in xenografted mice	(Guo et al., 2024a)
	Small nucleolar RNA host gene 5 (SNHG5)	*SNHG5* was ↑ expressed in spheroids, as compared to 2D monolayer cells. Silencing *SNHG5* ↓ spheroid formation and ↓ tumor growth, volume, and weight in xenografted mice. Spheroids were enriched in CSCs and had ↑ expression of *SNHG5*	(Li & Hu et al., 2022a)
	Small nucleolar RNA host gene 9 (SNHG9)	*SNHG9* was ↑ expressed in hepatocellular carcinoma tissues and cell lines, including CD133+ hepatocellular carcinoma CSC cells. Overexpressing *SNHG9* ↑ spheroid formation of CD133- cells. Silencing *SNHG9* ↓ spheroid formation of CD133+ cells, as well as tumor volume and weight in xenografted mice	(Yang et al., 2023b)
Lung cancer	ADAMTS9 antisense RNA 1 (ADAMTS9-AS1)	*ADAMTS9-AS1* was ↓ expressed in most cancer types (including lung adenocarcinoma), in both tissues and cell lines. Overexpressing *ADAMTS9-AS1* ↓ spheroid formation as well as tumor growth and weight in xenografted mice	(Wang et al., 2023a)
	ASAP1 intronic transcript 1 (ASAP1-IT1)	*ASAP1-IT1* was ↑ expressed in NSCLC tissues and cell lines. It was correlated with tumor differentiation, tumor node metastasis (TNM), and lymph node metastasis (LNM). *ASAP1-IT1* was also ↑ expressed in spheroids, as compared to 2D monolayer cells. Overexpressing *ASAP1-IT1* ↑ spheroid number. Silencing *ASAP1-IT1* ↓ spheroid formation and tumor growth in xenografted mice	(Liu et al., 2021a)
	GPAT4 and GINS4 antisense RNA 1 (GPAT4-AS1)	*GPAT4-AS1* ,also known as *GIAT4RA*, was ↓ expressed in lung tissues (both adenocarcinoma and squamous carcinoma) and associated with higher survival of patients. *GPAT4-AS1* was also associated with tumor size, clinical stage, and lymphatic metastasis. Overexpressing *GPAT4-AS1* ↓ spheroid size and number, as well as tumor growth and weight in xenografted mice. Silencing *GPAT4-AS1* had the opposite effects	(Yang et al., 2019b)
	Long intergenic non-protein coding RNA 1419 (LINC01419)	*LINC01419* was ↑ expressed in lung adenocarcinoma tissues and cell lines. Overexpressing *LINC01419* ↑ spheroid formation. Silencing *LINC01419* ↓ tumor growth, weight, volume, and proliferative levels in xenografted mice	(Chen et al., 2022a)
	Long intergenic non-protein coding RNA 467 (LINC00467)	*LINC00467* was ↑ expressed in lung adenocarcinoma tissues and cell lines, and high expression of *LINC00467* was associated with ↓ survival. Silencing *LINC00467* ↓ spheroid number and tumor size and weight, as well as ↓ proliferation and ↑ apoptosis levels in xenografted mice	(Chang & Yang, 2020)
	MNX1 antisense RNA 1 (head to head) (MNX1-AS1)	*MNX1-AS1* was ↑ expressed in lung tissues, including lung adenocarcinoma, and also in CSCs. Silencing *MNX1-AS1* ↓ spheroid formation of CSCs and the tumor growth rate and weight in xenografted mice	(Lv et al., 2023)
	OIP5 antisense RNA 1 (OIP5-AS1)	*OIP5-AS1* was ↑ expressed in lung cancer cells, and was also ↑ expressed in spheroids, as compared to 2D monolayer cultures. Silencing *OIP5-AS1* ↓ spheroid number and size. Overexpressing *OIP5-AS1* ↑ spheroid number and size, as well as tumor formation rates in *in vivo* experiments	(Mao & Li, 2022)
	SOX2 overlapping transcript (SOX2OT)	*SOX2OT* was ↑ expressed in lung tumors and resistant cell lines. Patients with high *SOX2OT* expression had ↓ survival. *SOX2OT* was also ↑ expressed in CSC-derived spheroids, as compared to 2D monolayer cells. CSCs isolated from spheroids had ↑ capacity to initiate solid tumor formation *in vivo*, as compared to 2D monolayer cells. Silencing *SOX2OT* ↓ spheroid formation and, when combined with GL1T repression, tumor growth in xenografted mice also ↓	(Dong et al., 2023)
	Testis development related 1 (TDRG1)	*TDRG1* was ↑ expressed in non-small cell lung cancer cells. Silencing *TDRG1* ↓ spheroid number and size. When inoculated into mice it ↓ tumor formation rate and stem cell frequency. Overexpressing *TDRG1* had the opposite effects	(Lu et al., 2023)
Medulloblastoma	LOXL1 antisense RNA 1 (LOXL1-AS1)	*LOXL1-AS1* was ↑ expressed in medulloblastoma tumors and cell lines. Patients with ↑ expressed *LOX1-AS1* had ↓ survival rates. *LOXL1-AS1* silencing ↓ spheroid formation, which was ↑ under *LOXL1-AS1* overexpression. Xenografted mice with *LOXL1-AS* overexpression had ↓ survival rates	(Do et al., 2024)
Oral squamous cell carcinoma	TINCR ubiquitin domain containing (TINCR)	*TINCR* was ↓ expressed in oral squamous cell carcinoma tissues, specifically in poorly differentiated tissues, and patients with low expression of *TINCR* had ↓ survival. *TINCR* was also positively correlated with the pathological differentiation stage. *TINCR* was also ↓ expressed in spheroids, as compared to 2D monolayers, a process that was associated with dedifferentiation. Overexpression of *TINCR* ↓ tumor size, weight, and volume in xenografted mice	(Z. Zhuang et al., 2021)
Osteosarcoma	B4GALT1 antisense RNA 1 (B4GALT1-AS1)	*B4GALT1-AS1* was ↑ expressed in osteosarcoma tissues, cell lines, and spheroids, as compared to 2D monolayer cells. Silencing *B4GALT1-AS1* ↓ spheroid size and number, and, in xenografted mice, ↓ tumor size, weight, seeding ability, and formation rate	(Li et al., 2018b)
	WAC antisense RNA1 (WAC-AS1)	*WAC-AS1* was ↑ expressed in most cancer types (including osteosarcoma), specifically, in metastatic tissues, as compared to non-metastatic and normal tissues. *WAC-AS1* expression was also correlated with lung metastasis, and patients with high expression of *WAC-AS1* had ↓ survival rates. Overexpressing *WAC-AS1* ↑ spheroid number, as well as tumor growth and metastasis in xenografted mice	(Yang et al., 2023c)
Ovarian cancer	HOX transcript antisense RNA (HOTAIR)	*HOTAIR* was ↑ expressed in ovarian cancer (specifically in stages I/II) and was correlated with tumor stage. Plasma circulating levels of *HOTAIR* showed high specificity and sensitivity as a diagnostic biomarker in ovarian cancer. *HOTAIR* was also ↑ expressed in spheroids, as compared to 2D monolayer cells. Silencing *HOTAIR* ↓ spheroid formation and the rate of tumorigenesis, as well as proliferation levels, in xenografted mice	(Y. Zhang et al., 2020)
Pancreatic cancer	DDIT4 antisense RNA 1 (DDIT4-AS1)	*DDIT4-AS1* was ↑ expressed in pancreatic ductal adenocarcinoma tissues and cell lines, and was also correlated with tumor size, tumor stage, and lower survival rates. Overexpressing *DDIT4-AS1* ↑ spheroid formation. Silencing *DDIT4-AS1* ↓ formation of both primary and secondary spheroids. Silencing *DDIT4-AS1* in cells injected into mice, and injecting the silencing machinery intratumorally, ↓ both tumor incidence, growth, and weight in xenografted mice. Silencing *DDIT4-AS1* and treating with gemcitabine further ↓ tumor incidence, growth, weight, and proliferation levels, and ↑ apoptosis levels, in xenografted mice	(Zhang et al., 2022c)
	Pvt1 oncogene (PVT1)	*PVT1* was ↑ expressed in spheroids, as compared to 2D monolayer cells. Curcumin treatment, alone or in combination with gemcitabine, ↓ *PVT1* expression in tumors, as well as tumor weight, in xenografted mice	(Yoshida et al., 2017)
Prostate cancer	MBNL1 antisense RNA 1 (MBNL1-AS1)	*MBNL1-AS1* expression was correlated with the stemness index, prognosis, tumor progression, recurrence, and drug resistance, and inversely correlated with immune responsiveness and immunotherapy effects. Silencing *MBNL1-AS1* ↑ spheroid formation from CSCs and ↑ proliferative and invasive abilities in xenografted mice	(J. Liu et al., 2022)
Wilms’ tumor	Long intergenic non-protein coding RNA 667 (LINC00667)	Silencing *LINC00667* ↓ spheroid formation from primary cells. Knocking-out *LINC00667* ↓ tumor growth, volume, and weight in xenografted mice	(Liu et al., 2020a)
	EMX2 opposite strand/antisense RNA (EMX2OS)	*EMX2O*S was ↓ expressed in Wilms’ tumor patients. Overexpressing *EMX2OS* ↓ the number of spheroids and tumor weight in xenografted mice	(Zhang et al., 2023a)

## Organoids: laboratory-made mini-organs

Organoids are miniature versions of organs or tissues. They are generated from biological materials containing adult or pluripotent stem cells through *in vitro* growth in the presence of ECM proteins and biochemical signals. These signals drive stem cell aggregation, self-organization, and differentiation into a physiologically active microscopical structure (Dutta et al., 2017; X. Liu et al., 2025; Yang & Hu et al., 2023a). When generated from cancer tissues, organoids can be termed tumoroids and provide a disease model of a specific cancer type, mimicking features of the native tumors and their respective patients (Guo et al., 2024b).

In the context of cancer, organoids are quite versatile to study the effects of lncRNA overexpression or silencing. For example, *lncRNA glioma radiation sensitizers 1* (*lncGRS-1*), which is upregulated in both adult and pediatric forms of glioma, was silenced in glioma organoids by CRISPR interference (CRISPRi) or antisense oligonucleotides (ASOs), decreasing their proliferation. Such silencing enhanced radiation sensitivity, further decreasing cancer cell proliferation in the organoid. Furthermore, organoids composed of healthy human astrocytes and glioma cells were prepared. In these organoids, *lncGRS-1* silencing was not harmful to the healthy astrocytes (Liu et al., 2020b), demonstrating the promising safety of this potential therapeutic approach. Of note, *lncGRS-1* orthologs do not exist in mice, chicken, or zebrafish, for which studies in animal models would not be feasible for this lncRNA. As such, 3D cellular models provide a viable alternative, with the advantage of being representative of the human species, unlike animal models. This study not only exemplifies organoids as additional 3D cellular models that can be used to assess the effects of lncRNA overexpression or silencing in cancer, but also accentuates the usefulness of 3D models in cases of poor conservation of lncRNAs in commonly used animal models.

### Patient-derived organoids can mimic *ex vivo* tumors

Several studies have compared the impact of lncRNA overexpression or silencing on organoids and xenograft mouse models, and some also included spheroids (Table 3). For example, in colorectal cancer, *POU6F2 antisense RNA 2* (*POU6F2-AS1*) silencing suppressed patient-derived organoid formation, while its overexpression had the opposite effect. *POU6F2-AS1* silencing also decreased cell growth, and tumor volume and weight in a xenograft model (T. Jiang et al., 2024). Additional studies also assessed metastasis in xenograft models. In gastric cancer, silencing of *C8orf76*, which directly binds and induces lncRNA *Dual specificity phosphatase 5 pseudogene 1* (*DUSP5P1*), suppressed the growth of patient-derived organoids and tumors in a xenograft mouse model. In mice, *C8orf76* silencing also suppressed lung and liver metastasis (X. Wang et al., 2019). These studies demonstrate that the effects of lncRNA overexpression or silencing are consistent between organoids and tumors representing the same cancer type. Furthermore, animals provide additional information on metastasis formation.

**Table 3:** Studies that focus on the combined use of organoids and xenografts

Cancer type	lncRNA	Summary	Ref
Breast cancer	DDIT4 antisense RNA 1 (DDIT4-AS1)	*DDIT4-AS1* was ↑ expressed in triple negative breast cancer tissues and cell lines, and was positively associated with the TNM stage, Ki67 proliferation marker, and patient survival. *DDIT4-AS1* was dysregulated by autophagy induction. Simultaneous silencing of *DDIT4-AS1* and paclitaxel treatment ↓ patient-derived organoid quantity, size, and cell viability, as well as tumor growth and proliferation levels in xenografted mice	(Jiang et al., 2023)
	Metastasis-associated lung adenocarcinoma transcript 1 (MALAT1), nuclear paraspeckle assembly transcript 1 (NEAT1)	*MALAT1* was ↑ expressed in lung metastasis cancer cells (as compared to matched breast cancer primary tumors) and in advanced carcinomas (as compared to adenomas or hyperplasia). Silencing *MALAT1* ↓ organoid size, branching, cellular motility, and collective cell migration, ↑ organoid compaction, and organized organoids in a polarized, differentiated manner, similar to normal mammary glands. Delivery of *MALAT1* to *MALAT1* knockout organoids rescued the branching to the same level of wild-type organoids. *MALAT1* knockout mice developed cystic-filled tumors and had ↓ metastatic burden, as compared to controls consisting of poorly differentiated solid carcinomas. Silencing *NEAT1* resulted in similar branching alterations to controls, but there was ↑ apoptotic cell death and ↓ in the number of dividing cells	(Arun et al., 2016)
	PDCD4 antisense RNA 1 (PDCD4-AS1)	*PDCD4-AS1* was ↓ expressed in triple-negative breast cancer tissues, as compared to other breast cancer subtypes, specifically in the most aggressive stages. High expression of *PDCD4-AS1* was correlated with ↑ patient survival rates. Normal breast cells were modified and selected from xenografts to be increasingly aggressive and were then cultured in Matrigel as organoid-like structures, where *PDCD4-AS1* was ↓ expressed in the most aggressive stages of breast cancer	(Jadaliha et al., 2018)
Cholangiocarcinoma	TTN antisense RNA 1 (TTN-AS1)	*TTN-AS1* was ↑ expressed in cholangiocarcinoma tissues, circulating tumor cell exosomes, and blood. High expression of *TTN-AS1* was associated with LNM and TNM. Organoids were established using circulating tumor cells of patients, and, in exosomes from their extracellular vesicles (EVs), *TTN-AS1* was ↑ expressed. Such exosomes were collected from *TTN-AS1* overexpressing organoids and co-cultured with cholangiocarcinoma cells, which were then injected into mice. *TTN-AS1* overexpression in organoids resulted in downstream ↑ tumor volume, weight, and proliferation levels in xenografted mice, while silencing *TTN-AS1* in organoids had the opposite effect	(Zhou et al., 2024a)
Colorectal cancer	Maternally expressed 3 (Meg3)	*Meg3* was ↓ expressed in colorectal cancer tumors. *Meg3* accumulated in the colonic crypts of tumors in xenografted mice and organoids were derived from colon cells of these mice. Silencing *Meg3* in mice resulted in downstream ↑ organoid growth, while *Meg3* overexpression ↓ organoid growth	(Zhang et al., 2022b)
	POU6F2 antisense RNA 1 (POU6F2-AS1)	*POU6F2-AS1* was ↑ expressed in colorectal cancer tumors and cell lines and was associated with tumor diameter, depth of invasion, the LNM and TNM stages, and ↓ survival. Overexpressing *POU6F2-AS1* ↑ patient-derived organoid formation. Silencing *POU6F2-AS1* ↓ patient-derived organoid formation and ↓ volume, weight, proliferative levels, and lipid content of xenografted mouse tumors	(T. Jiang et al., 2024)
	Small Cajal body-specific RNA 2 (SCARNA2)	*SCARNA2* was ↑ expressed in radioresistant patients, and patients with high expression of *SCARNA2* had ↓ survival. Silencing *SCARNA2* combined with radiotherapy ↓ patient-derived organoid growth, as well as tumor weight and progression in xenografted mice. Delivery of *SCARNA2* silencing machinery directly to xenografted mice also ↓ tumor growth, weights, and proliferative levels, and ↑ apoptotic levels	(Y. Chen et al., 2023)
Gastric cancer	DSP antisense RNA 1 (DSP-AS1)	*DSP-AS1*, also known as *ABL*, was ↑ expressed in gastric cancer tumors and cell lines and was a predictive marker for gastric cancer prognosis. *DSP-AS1* was also associated with ↓ survival of patients. Overexpressing *DSP-AS1* ↑ organoid diameter, cell growth, and ↓ cisplatin/paclitaxel-induced apoptosis. Overexpressing *DSP-AS1* in xenografted mice ↑ size, weight, growth, proliferative levels, and ↓ the number of apoptotic cells and thus cisplatin effect. Co-delivering silencing machinery with paclitaxel in xenografted mice ↓ tumor size, weight, growth, proliferative levels, and the number of apoptotic cells	(Wang et al., 2022b)
	Dual specificity phosphatase 5 pseudogene 1 (DUSP5P1)	In xenografted mice, overexpressing *DUSP5P1* ↑ tumor growth, numbers of proliferating cells and ↓ numbers of apoptotic cells. Silencing of *C8orf76*, which directly binds and induces *DUSP5P1*, ↓ the growth of patient-derived organoids and xenografted mice	(X. Wang et al., 2019)
Glioma	lncRNA glioma radiation sensitizers 1 (lncGRS-1)	Glioma cells were seeded on the surface of normal brain organoids, which the cancer cells invaded and transformed into tumors. *LncGRS-1* silencing reduced tumor growth and sensitized it to radiotherapy, without affecting the healthy organoids	(Liu et al., 2020b)
	MIR22 host gene (MIR22HG)	*MIR22HG* was ↑ expressed and transcriptionally active in glioblastoma, as well as in its subtype with worst outcomes, especially in the infiltrating regions of the tumor, as compared to the core. Patients with high *MIR22HG* expression had ↓ survival, and *MIR22HG* was also validated as an independent prognostic indicator of patient survival. Silencing *MIR22HG* ↓ formation and the invading distance of spheroids. When co-cultured with brain organoids, silencing *MIR22HG* in spheroids ↓ the number of invading tumor cells. Silencing *MIR22HG* in xenografted mice ↓ growth, proliferative, and invasive markers and ↑ survival of tumor-bearing mice	(M. Han et al., 2020)
Hepatocellular carcinoma	BBOX1 antisense RNA 1 (BBOX1-AS1)	*BBOX1-AS1* was ↑ expressed in hepatocellular carcinoma cell lines and tumors, especially in patients with vascular invasion and advanced TNM stage. High expression of *BBOX1-AS1* was associated with ↓ survival. *BBOX1-AS1* was also validated as an independent risk factor for hepatocellular carcinoma. In intermediate resistance organoids, silencing *BBOX1-AS1* ↓ organoid growth, which was enhanced when coupled with sorafenib treatment. Silencing *BBOX1-AS1* and treating with sorafenib ↓ tumor growth and proliferative levels in xenografted mice. Patient-derived organoids were evaluated for sorafenib IC50 and the most resistant organoids had ↑ expressed *BBOX1-AS1* as compared to the less resistant. Overexpressing *BBOX1-AS1* ↑ tumor size, proliferative levels, and the number of metastatic nodules in xenografted mice	(Tao et al., 2023)

Several studies have also assessed the effects of lncRNA overexpression or silencing on the organoid response to chemo- or radiotherapy. In gastric cancer, overexpression of *DSP antisense RNA 1* (*DSP-AS1*) was associated with larger organoid dimensions and increased tumor size in mice. Furthermore, organoids with overexpressed *DSP-AS1* were more resistant to cisplatin/paclitaxel-induced apoptosis (Wang et al., 2022b). In rectal cancer, silencing of *small Cajal body-specific RNA 2* (*ScaRNA2*) increased sensitivity to radiotherapy on patient-derived organoids and xenograft models (Y. Chen et al., 2023). In triple-negative breast cancer, silencing of *DNA damage inducible transcript 4 antisense 1* (*DDIT4-AS1*) enhanced the efficacy of paclitaxel on patient-derived organoids and on a xenograft mouse model (Jiang et al., 2023). These studies further demonstrate that lncRNA silencing may also increase organoid sensitivity to chemo- or radiotherapy.

Organoids can also be obtained from xenografted animals instead of patients. *Maternally expressed gene 3* (*Meg3*) was overexpressed or silenced in xenografted mice. The xenografts were further used to generate organoids. In both xenografts and xenograft-derived organoids, *Meg3* silencing or overexpression increased or decreased their size, respectively (S. Zhang et al., 2021).

Importantly, organoids are gold standards of “personalized” medicine, reflecting personal aspects of individual patients. In hepatocellular carcinoma, organoids derived from different patients had slightly different levels of sorafenib resistance. Sorafenib and *BBOX1 antisense RNA 1* (*BBOX1-AS1*) silencing inhibited organoid growth, with synergistic effects. On the other hand, *BBOX1-AS1* overexpression in a xenograft model increased tumor size and metastasis (Tao et al., 2023).

While xenograft models are necessary to assess metastasis and survival rates, patient-derived organoids have the advantage of better reflecting human patients’ features. The above-mentioned studies suggest that xenograft mouse models might be replaced with cognate organoids if only tumor growth or an indicator of therapy response needs to be assessed.

## Advanced 3D cellular models: underexplored alternatives?

While spheroids and organoids have been extensively used in lncRNA cancer research, other 3D cellular models, specifically microfluidic systems such as “organs-on-chips”, have been less exploited in this framework. Microfluidic systems are devices that connect several channels between wells, where each well is filled with cells from the organ they represent. Immune and tumor cells can circulate through these channels and thus, any associated immune response can be monitored (Li et al., 2024a).

In the context of triple-negative breast cancer, *H19* was studied in microfluidic-based 3D devices that allow co-cultures and hypoxia studies. Because MSCs play an important role in mediating cancer cell invasion, they were co-cultured in microfluidic devices with breast cancer cells. In hypoxic conditions, MSCs had enhanced migration and guided breast cancer cells in a “cluster-sprout-infiltrating” way relative to normoxic conditions. In this study, *H19* was induced and upregulated in both hypoxic conditions and in a time-dependent manner. Silencing *H19* did not change the morphology of MSCs, but delayed their sprouting, infiltration, and reduced their migration distance. These outcomes impaired and retarded cancer cell migration. Interestingly, under hypoxic conditions, aspirin treatment downregulated *H19* in MSCs, in a time-dependent manner. These effects were similar to shRNA-mediated silencing of *H19* (An et al., 2022). Microfluidics can thus recreate cell intravasion events on the endothelial barrier of tumors, with precise control of growth-factor gradients and tunable endothelial permeability (Zervantonakis et al., 2012). Another study embedded spheroids in a microfluidic device and studied the effect and rate of internalization of lncRNA *RNA component of 7SK nuclear ribonucleoprotein (RN7SK)*-loaded exosomes. The lncRNA was synthesized and loaded into exosomes that were placed into side channels, and the spheroids were injected within a hydrogel in the central channel. The uptake of the exosomes led to an overexpression of *RN7SK* in the non-small cell lung cancer cells, whose viability was reduced, and resulted in the suppression of spheroid dispersion, mimicking an inhibition of the initial metastatic epithelial-mesenchymal transition step (Aghamiri et al., 2026). This shows that microfluidic devices are exceptionally well suited to study migration dynamics of cancer cells responding to both exosomes and cellular cues.

There are also 3D organotypic cultures, such as laminin-rich ECM-based three-dimensional organotypic (lrECM 3D) cultures, where cells were seeded in Matrigel-coated plates, in which *HOTAIR* and its isoforms were studied. *HOTAIR-N* was found to be a major isoform in invasive carcinoma. Its upregulation in triple-negative breast cancer increased with culture time, and both *HOTAIR* and *HOTAIR-N* were upregulated in 3D conditions, as compared to 2D cultures. Silencing *HOTAIR* decreased invasive growth patterns, cell numbers, and viability. Furthermore, silencing or inhibition of integrin α2, Src, or BRD4 in 3D conditions resulted in downregulated *HOTAIR*, thus showing that manipulation of ECM-related proteins can impact lncRNAs (M. Li et al., 2017).

These examples demonstrate that increasing the complexity of 3D cellular models may bring further insights into our knowledge of the impact of lncRNAs in cancer. The increased complexity of these 3D cellular models could also contribute to increasing their capability of replacing animal models in research. Nevertheless, assessing such capability requires further and extensive research using these advanced 3D cellular models.

## Discussion

In the present review, we analyzed a representative sample of original research studies involving at least one 3D cellular model to assess the impact of lncRNA overexpression or silencing in different types of cancer. The most used 3D model was the spheroid, followed by the organoid. Many of these studies also involved an animal model, which, in most cases, was a mouse xenograft. In most of these studies, a large component of experimental work was carried out in 2D cellular cultures. However, we focused only on the experimental data obtained in 3D and animal models, since those were the most relevant to our review. Since the number of relevant studies is very high (and permanently increasing), we could not perform an exhaustive analysis. As such, most articles chosen for analysis were published between 2022 and 2025. To ensure a representative literature sample, the number of analyzed studies involving each 3D model or 3D+ animal model combination was proportional to the respective abundance within the literature. Our understanding was also limited by the level of detail provided in the methods sections of the original research publications.

### lncRNAs are disease context-dependent

In our literature search, multiple solid cancer types were addressed. The breast, colorectal, lung, liver, and brain cancers were the most found. Overall, more than half of the studied lncRNAs were sense, being *H19* and *HOTAIR* the most studied lncRNAs, followed by *MALAT1*, *PVT1*, and *LINC-ROR*. In most studies, lncRNA levels were overexpressed or silenced (with or without drug testing) before assembly of the 3D *in vitro* cellular model. We also noticed that the vast majority of the studies focused on lncRNAs with oncogenic or tumor-promoting functions that are naturally upregulated in the tumors, which were then experimentally manipulated through gene silencing. For correlative studies of lncRNA expression, most studies used databases, such as GENCODE, ENCODE, and GEO, which contain gene annotations and gene expression repositories (Pinkney et al., 2020). Some of them also analyzed a new cohort as an additional validation of their results.

Because lncRNA function and impact are context-dependent, each lncRNA may have different effects in different cancer types. For example, silencing *H19* in either colorectal cancer (M. Zhu et al., 2024) or papillary thyroid carcinoma (Li et al., 2018a) decreased spheroid formation capacity; however, in breast cancer, silencing this lncRNA increased spheroid formation (Shima et al., 2018). Another example is *HIF2PUT*, whose silencing decreases colorectal cancer spheroids formation and self-renewal capabilities (Yao et al., 2015). However, in osteosarcoma, its silencing resulted in increased spheroid formation rates (Y. Wang et al., 2015). In glioma tissues, *LINC-ROR* was downregulated and its overexpression decreased spheroid number (Feng et al., 2015). Furthermore, in esophageal squamous cell carcinoma tissues, *LINC-ROR* was, on the other hand, upregulated and its silencing decreased both the formation of spheroids and tumors in xenografted mice (L. Wang et al., 2017). These examples demonstrate the complexity of lncRNA mechanisms in cancer and spheroid formation. Therefore, any generalization across cancer types should be carefully considered.

Although the same lncRNA can have different impacts in different cancer types, its response in 3D and animal models of the same cancer type is quite consistent. We found only one study with potentially conflicting results between a 3D and an animal model. In breast cancer, *LINC00052* expression decreased upon spheroid formation. However, its silencing failed to affect spheroid formation, but enhanced cell migration in a zebrafish xenograft model (Sanchez-Lopez et al., 2021). Of note, the phenotypic processes studied in spheroids and zebrafish were not exactly the same, which could explain the different results. Most studies used only one cell line; however, using more cell lines would strengthen the generalizability of the results for their respective cancer type. Some studies compared expression levels between 2D and 3D models (Li et al., 2017). However, in 3D cellular models, most studies did not test any additional hypotheses beyond lncRNA overexpression/silencing or drug resistance, revealing some gaps and opportunities to explore in future research.

### Manipulation of lncRNA expression

Several systems were used to manipulate lncRNA expression, most of them employed in monolayer cultures preceding both 3D assembly and xenograft injection. For example, for silencing, CRISPR/Cas genome editing was widely used for permanent knockout. This process can be challenging, as the editing efficiency can be low, the selection and optimization processes can be time-consuming, and the outcomes may compromise cell survival (if homozygotic) and bring off-target and unexpected outcomes. On the other hand, CRISPRi recruits transcriptional repression factors to the gene promoter, decreasing RNA transcription levels, which provides a less drastic effect than the knockout (Ghavami & Pandi, 2021).

For transient knockdown of lncRNAs in 3D cellular models, two possibilities are small interfering RNA (siRNA) and short hairpin RNA (shRNA). An siRNA consists of a double-stranded RNA molecule that is chemically synthesized and transiently transfected into target cells, where it activates a protein complex that degrades target RNA. The same RNA-degrading mechanism can be activated through shRNA, in which target cells are transfected with a vector that expresses a single RNA molecule that self-hybridizes (generating a short hairpin-like structure) to mimic the siRNA duplex. While both siRNA and shRNA activate the same RNA degradation mechanism, siRNA is adequate for the degradation of cytoplasmic RNAs over short time periods, since it is delivered as RNA, into the cytoplasm, where its lifetime is short. On the other hand, shRNA is delivered as its expression vector, being continuously transcribed in the nucleus. Therefore, shRNA usually provides longer-lasting RNA degradation than siRNA and can degrade nuclear RNA. Nevertheless, if a prolonged siRNA effect is needed, repeated transfection may be necessary, and penetration into the core of already formed 3D structures can be very limited. An additional approach consists of ASOs, which are single-stranded DNA molecules that hybridize with target RNA, inducing its degradation by RNAse H. ASOs can be chemically modified to increase their binding affinity and lifetime. Since RNAse H can be found in both the nucleus and cytoplasm, ASOs can degrade both nuclear and cytoplasmic lncRNAs. Due to the enhanced stability of DNA relative to RNA, ASOs can provide longer-lasting effects than siRNA. (Lennox & Behlke, 2016; S. Zibitt et al., 2021). Thus, the choice of an RNA silencing approach could be guided by the subcellular location of the target lncRNA and the duration of the intended effect.

For lncRNA overexpression in 3D cellular models, their endogenous transcription can be increased by CRISPR activation, which uses a CRISPR-associated protein to recruit transcriptional activators to the promoter (Chavez et al., 2016). Alternatively, lncRNA can be transcribed from an exogenous expression vector. The vector can be delivered to the cells by lipofection, electroporation (electrical pulses), or packaged inside artificial viruses. If an exogenously expressed lncRNA is expected to have a nuclear location, an appropriate nuclear retention signal should be added to its sequence (Wei et al., 2023). The choice of a specific lncRNA overexpression approach should be guided by the efficiency and homogeneity of the effect needed. Electroporation and viral infection usually result in higher percentages of expressing cells, with more homogeneous expression levels, relative to lipofection.

Both organoids and mouse models contain multiple cell types, requiring efficient delivery of molecules for lncRNA manipulation and drugs to the intended cell type. One strategy for targeted delivery is the encapsulation of drugs inside polymeric or vesicular nanoparticles coated with ligands recognized by receptors upregulated on cancer cells. Fine-tuning of nanoparticle dimensions prevents renal filtration and phagocytosis. SiRNAs can also be delivered alongside therapeutic drugs (Ciftci et al., 2025), being convenient for use in animal models. Targeted delivery has several advantages over conventional delivery: it increases the solubility, chemical stability, and half-life of the drug. Furthermore, specific targeting allows delivery to the target site at better controlled rates and reduces its systemic distribution, decreasing any side effects (Tewabe et al., 2021). Furthermore, the use of expression vectors with cell type-specific promoters (Zheng & Baum, 2008) could enable lncRNA overexpression only in specific tissues. Thus, targeted drug delivery should be preferred in both organoid and animal research.

### Spheroids: simple 3D models

In most reviewed studies, spheroids were simple, homotypic, and assembled in ULA plates, without ECM, perfusion, or co-culture conditions. Nevertheless, they generally showed a decreased and more heterogeneous response to chemotherapeutic drugs relative to 2D-cultured cells. This critical difference increases the similarity of the spheroid to a real tumor, with the advantage of being straightforward and inexpensive to generate. Some studies injected spheroids (or cells obtained from spheroids) directly into mice (Wang et al., 2020), while others used cells from mouse xenografts to generate spheroid. Furthermore, some studies used primary cell lines from patients to generate spheroids. Some studies even correlated therapy resistance in spheroid and/or animal models with the patient’s respective response. It was observed that overexpressing or silencing specific lncRNAs could modulate the efficiency of formation and dimensions of spheroids. Of note, the effects on spheroid size were consistent with those observed in mouse xenografts.

Despite the versatility of spheroids as 3D cellular models, most of the reviewed studies only tested whether lncRNA overexpression or silencing affected spheroid formation, assessed as spheroid number and size (through a spheroid formation assay [SFA]). In these studies, increased stemness was associated with increased spheroid formation efficiency. Only a relatively small fraction of these studies utilized spheroids for testing experimental hypotheses, such as variations in drug sensitivity. In general, no additional experiments were performed using these 3D cellular systems. As such, most studies involving spheroids did not fully exploit this model to further study lncRNA function, interactions, localization, or any other properties. For example, many of these studies did not perform drug testing in 3D *in vitro* models to assess the potential of lncRNA manipulation toward a neoadjuvant therapy. Nevertheless, in future studies, their complexity could be further increased by introducing more cell types, distinct matrix components, or even perfusion; therefore, increasing their similarity to native tumors. Future studies using spheroids should take advantage of compatible techniques, including lncRNA sequencing using ribosome-bound RNA (Cornelissen et al., 2024), RNA-seq and single-cell RNA-seq (Yoon et al., 2020), or time-course visualization of protrusions/detached clusters and associated gene expression patterns (Li et al.,2024c).

### Organoids: more complex 3D models

Organoids are more complex 3D cellular models than spheroids, with the advantage of being able to better reflect patient features, such as their personal level of resistance to chemotherapy. Unlike spheroids, a significant percentage of the studies involving organoids tested variations in radiation or drug sensitivity associated with lncRNA overexpression or silencing. As in spheroids, organoids also revealed consistent responses to lncRNA overexpression or silencing relative to mouse xenografts, which provides robustness to *in vivo* conclusions.

The value of organoids is strengthened by their compatibility with advanced technologies. Differences in lncRNA expression levels in different cells (or subcellular locations) of the same organoid can be assessed using spatial transcriptomics (Williams et al., 2022). This approach is broadly divided into imaging-based (visualization of single RNA molecules by fluorescence microscopy) and sequencing-based (identification of RNA sequences isolated from specific cells or subcellular locations) technologies. A common requirement is the isolation of intact and viable cells, which may be technically challenging. In addition to profiling lncRNA expression, identifying novel lncRNAs that affect organoid phenotype is another important goal. CRISPR screenings (Y. He et al., 2026) are a powerful approach that uses CRISPR-associated proteins to mutate or affect the expression of multiple genes (using multiple corresponding guide RNAs), to uncover those associated with cell proliferation or drug sensitivity/resistance. CRISPR screenings could be used to uncover lncRNAs with therapeutic potential in cancer, using 3D cellular models. They may also be further adapted for lncRNA silencing or overexpression. For example, *lncGRS-1* was identified through a CRISPR screening, which was further adapted for its silencing in organoids (Liu et al., 2020b). Thus, spatial transcriptomics and CRISPR screenings are innovative resources that enhance the capabilities of organoids and their potential to decrease animal use in research.

Organoids could be further used to test additional properties, such as branching morphogenesis, apoptosis (Arun et al., 2016), exosome transcriptome sequencing (Zhou et al., 2024a), and *in situ* RNA detection methods that allow the visualization of the spatial distribution of gene expression patterns (Liu et al., 2021b). These examples demonstrate that these 3D models are compatible with the study of multiple parameters and the use of various techniques, which is an advantage for their capacity to replace animal models. We found a larger number of studies combining organoids with mouse xenografts than studies using organoids solely. This difference reflects the necessity of animal models to assess parameters that cannot be assessed by organoids, such as metastasis and animal survival. Nevertheless, their combined use allows assessing how they complement each other and identifying some of the aspects in which the animal model might be (at least in part) replaced with a cognate organoid.

### *In vitro* 3D and animal models: advantages, limitations, and possibilities

Unlike animal models, both spheroids generated from human cells and patient-derived organoids are representative of the human species, providing ideal models of human diseases. They are critically important in studies of lncRNAs that are not conserved in mouse or other animal species, such as *lncGRS-1*. Spheroids and organoids are also advantageous relative to animal models for the study of gene mutations, considering the laborious process of generating genetically modified animal models. In Table 4, we provide a summary list of advantages and limitations of spheroids and organoids. As listed in Table 4, spheroids and organoids have intrinsic limitations, including the lack of a full immune system, as well as the absence or very incomplete stromal and vascular structures. Thus, studying the complexity of animal tumors and associated properties, such as the tumor microenvironment and angiogenesis, is limited in 3D cellular models. 3D cellular models can also have significant batch variability and be affected by variations in culture conditions, with impacts on reproducibility and standardization. The time and cost requirements associated with 3D cellular models, as well as their technical complexity, can be additional limitations. However, animal models also have considerable costs and requirements associated. In the near future, the development and commercial availability of ready-to-use 3D cellular models should contribute to decreasing their overall costs and technical challenges. Of note, the technical challenges of generating genetically modified animal models have decreased since biotechnological companies have started selling these resources. A similar outcome could be possible regarding 3D cellular models.

**Table 4:** Comparative assessment of 3D and animal models for lncRNA study

Criterium	**3D cancer cellular models (spheroids and organoids)**	**Animal models**
Ethics requirements	Simple, required mainly if using patient cells	Mandatory, as well as specific training
Monetary cost	Spheroids are usually cheaper to prepare than organoids	Animal models have higher costs than 3D cellular models
Representativeness of human patients	Yes. Any desired cell line may be used—human or animal, immortalized or primary. Organoids are more representative of the supra-cellular organization of organs, than spheroids	Limited
Manipulation of lncRNA levels and fine-tuning of its delivery	Straightforward, direct, several approaches available	More challenging to achieve in the whole organism or in specific body parts
Visualization of lncRNA spatial location	Limited by cellular density	Usually requires animal sacrifice, organ removal, and further processing
Feasibility of achieving statistically significant data	Assays in multiwell plates can simultaneously provide sufficient biological replicates for statistically robust conclusions	The use and sacrifice of multiple animals is required for statistically robust results
Immune response testing	Limited; immune cells can be added and their response assessed	Unrestricted, but with limited representativeness of the human species
Assessment of the extracellular matrix and tumor microenvironment	Limited; spheroids are more customizable in both stiffness, chemical components, and cells, as compared to organoids	Unrestricted
Variability among biological replicates	Dependent on the experimental assay, but possibly lower than in animal models	Significant variability among genetically identical animals
Assessment of lncRNA manipulation effect on the size of the “tumor”	Identical	

**Fig. 1:**
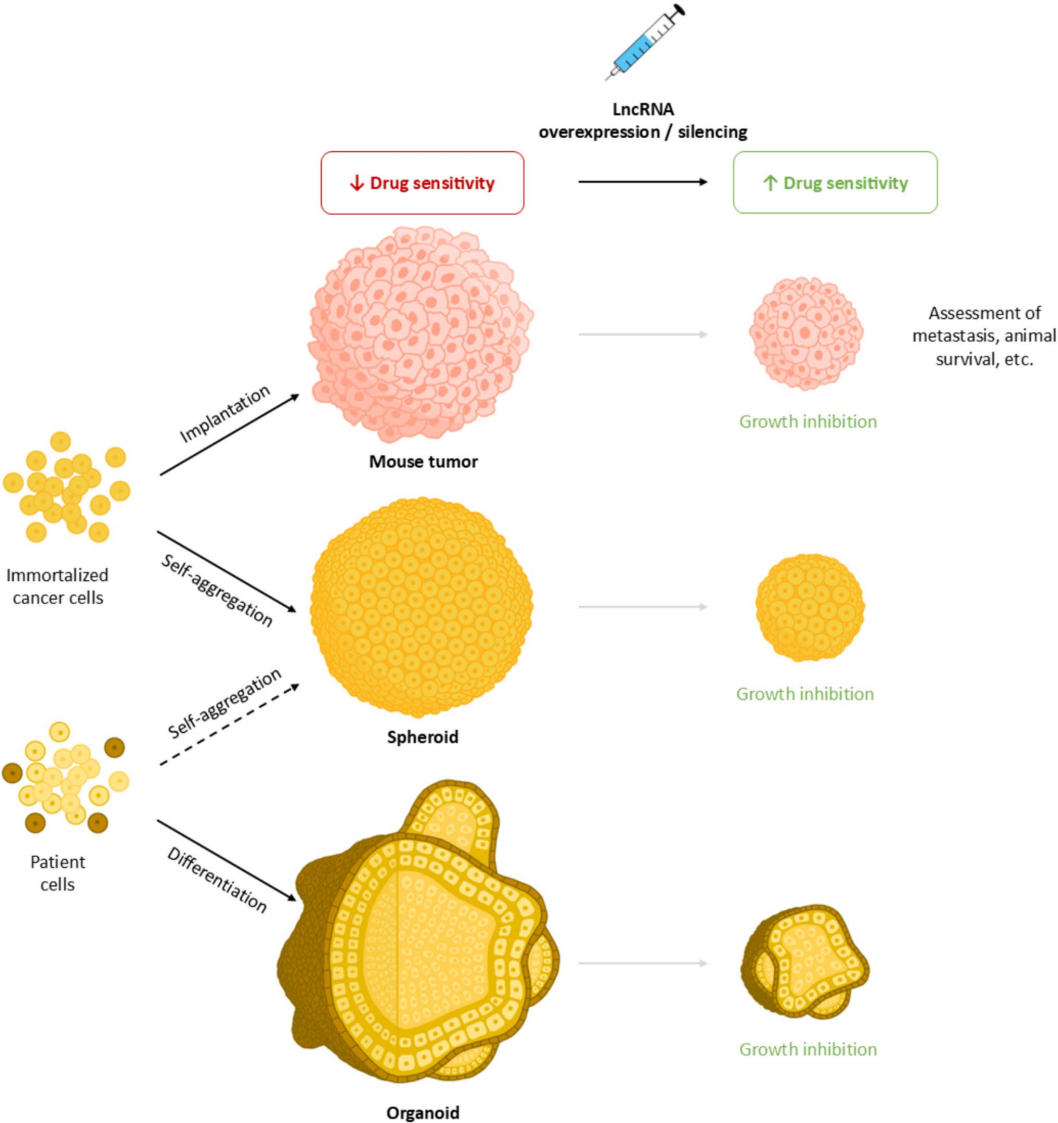
Cancer spheroids and patient-derived organoids can mimic the response of mouse tumors to long noncoding RNA (lncRNA) overexpression or silencing. Immortalized cancer cells can be used to generate spheroids or mouse tumors, through self-aggregation or their implantation in mice, respectively. Organoids can be generated from any patient cells through their differentiation, and, less frequently, patient cancer cells can self-aggregate into spheroids *in vitro*. Cancer spheroids, mouse tumors, and patient-derived organoids generally share a common feature: the overexpression or silencing of specific lncRNAs inhibits their growth and enhances their drug sensitivity. While spheroids and organoids suffice to mimic tumor regression, animal models remain necessary to monitor *in vivo* parameters, including metastasis and survival rates. Original illustration created by the authors

Concerning the xenograft studies, we noted that, in many of them, only tumor growth was assessed. Only a fraction of these studies assessed metastasis, animal survival rates, or any additional *in vivo* parameters. As such, not all xenograft research took full advantage of their animal models, providing results that could have been mimicked using spheroids, organoids, or other 3D cellular systems. Overall, our literature survey has uncovered that spheroids, organoids, and mouse xenografts have similar responses to lncRNA overexpression or silencing (Fig. 1).

### Potential to replace animal models

We propose that spheroids and organoids could fully replace xenograft animal models in studies in which only tumor growth is assessed. The use of co-cultures would also partially allow the study of the immune response (a common reason for choosing animal models), which would be important in the study of immune-related lncRNAs. Furthermore, future developments and upgrades in 3D cellular models are likely to decrease even further the need for animal models in biomedical research. The combination of spheroids with organoids could be one such development, which could allow testing invasive properties associated with cancer. However, in a specific study, brain-derived organoids (not composed of cancer cells) were co-cultured with glioblastoma spheroids. The goal was to evaluate the extent to which the cancer cells from the spheroid would (or not) metastasize to the organ. Parameters including the number of invading cancer cells and invaded organoid area were measured (M. Han et al., 2020). Therefore, the combination of the two 3D models enables the assessment of properties that cannot be assessed separately in one 3D model. Furthermore, microfluidic 3D cellular systems are still poorly explored in lncRNA cancer research and could be further used. For example, in breast cancer, a microfluidic 3D co-culture system was used to assess cancer cell migration under hypoxia. In this study, *H19* silencing decreased cell migration, which was mediated by *H19* and hypoxia (An et al., 2022). Specifically, this microfluidic 3D cellular model allowed the assessment of hypoxia effects. Therefore, more advanced 3D cellular models are likely to further decrease the need for animal models.

In addition to decreasing the use of mice, non-rodents such as zebrafish could be further used. Zebrafish xenograft models could provide similar results when compared with 3D cellular models and evaluate the same features and properties in a shorter time period and for a lower material investment (Y. Liu et al., 2023). As a representative study, in a zebrafish xenograft, the *long intergenic non-protein coding RNA 52 (LINC00052)* was shown to be downregulated when breast cancer formed spheroids; *LINC00052* downregulation increased cell migration in xenografted zebrafish embryos (Sanchez-Lopez et al., 2021). Further research should uncover the extent to which mice could be replaced with zebrafish as an animal model.

In the present review, we focused on lncRNAs in cancer research. Our findings are likely to apply to other types of RNA (such as messenger RNAs or micro-RNAs) and possibly to other biomedical research fields in which animal models are involved, such as metabolism and/or neuroscience.

## Data Availability

Data availability: not applicable.
